# Exercise-Induced Changes in Caveolin-1, Depletion of Mitochondrial Cholesterol, and the Inhibition of Mitochondrial Swelling in Rat Skeletal Muscle but Not in the Liver

**DOI:** 10.1155/2016/3620929

**Published:** 2015-12-29

**Authors:** Damian Jozef Flis, Robert Antoni Olek, Jan Jacek Kaczor, Ewa Rodziewicz, Malgorzata Halon, Jedrzej Antosiewicz, Michal Wozniak, Rosita Gabbianelli, Wieslaw Ziolkowski

**Affiliations:** ^1^Department of Bioenergetics and Physiology of Exercise, Medical University of Gdansk, 80-210 Gdansk, Poland; ^2^Department of Bioenergetics and Nutrition, Gdansk University of Physical Education and Sport, 80-336 Gdansk, Poland; ^3^Department of Physiotherapy, Gdansk University of Physical Education and Sport, 80-336 Gdansk, Poland; ^4^Department of Physiotherapy, Medical University of Gdansk, 80-210 Gdansk, Poland; ^5^Department of Biochemistry, Gdansk University of Physical Education and Sport, 80-336 Gdansk, Poland; ^6^Department of Medical Chemistry, Medical University of Gdansk, 80-210 Gdansk, Poland; ^7^School of Pharmacy, University of Camerino, 62032 Camerino, Italy

## Abstract

The reduction in cholesterol in mitochondria, observed after exercise, is related to the inhibition of mitochondrial swelling. Caveolin-1 (Cav-1) plays an essential role in the regulation of cellular cholesterol metabolism and is required by various signalling pathways. Therefore, the aim of this study was to investigate the effect of prolonged swimming on the mitochondrial Cav-1 concentration; additionally, we identified the results of these changes as they relate to the induction of changes in the mitochondrial swelling and cholesterol in rat skeletal muscle and liver. Male Wistar rats were divided into a sedentary control group and an exercise group. The exercised rats swam for 3 hours and were burdened with an additional 3% of their body weight. After the cessation of exercise, their quadriceps femoris muscles and livers were immediately removed for experimentation. The exercise protocol caused an increase in the Cav-1 concentration in crude muscle mitochondria; this was related to a reduction in the cholesterol level and an inhibition of mitochondrial swelling. There were no changes in rat livers, with the exception of increased markers of oxidative stress in mitochondria. These data indicate the possible role of Cav-1 in the adaptive change in the rat muscle mitochondria following exercise.

## 1. Introduction

The significance of the exercise-induced depletion in the mitochondrial cholesterol pool is still not fully understood. The reduction in cholesterol in rat heart mitochondria, observed after exercise, is related to the inhibition of mitochondrial swelling; however, this change does not influence the mitochondrial bioenergetics and oxidative stress. [1, 2]. It has been proposed that this phenomenon may be involved in the protection mechanism of mitochondria during exercise or during other stressful conditions [2].

Mitochondrial swelling [3], a significant mediator of cell death, results from the opening of a mitochondrial permeability transition pore (mPTP) [4, 5]. Important factor implicated in mPTP regulation is intracellular calcium. Physiological stimuli, such as physical exercise, that cause an increase of cytosolic free Ca^2+^ or the release of Ca^2+^ from the endoplasmic reticulum invariably induce mitochondrial Ca^2+^ uptake, with a rise of mitochondrial matrix-free Ca^2+^. Hence, mitochondria accumulate Ca^2+^ and efficiently control the spatial and temporal shape of cellular Ca^2+^ signals. This situation exposes mitochondria to the hazards of Ca^2+^ overload, which can lead to the opening of the mPTP. Persistent mPTP opening is followed by depolarization, with Ca^2+^ release, cessation of oxidative phosphorylation, matrix swelling with inner membrane remodelling, and, eventually, outer membrane rupture with release of cytochrome c and other apoptogenic proteins [6].

Caveolins (Cav) are essential components of caveolae, which are plasma membrane invaginations that demonstrate reduced fluidity; this reflects an accumulation of cholesterol [7]. Cav proteins bind cholesterol, and Cav's ability to move between cellular compartments helps to control intracellular cholesterol fluxes [7–9]. The first member of the Cav family (including Cav-1, Cav-2, and Cav-3), Cav-1, is a 22 kDa protein of 178 amino acids that plays an essential role in the regulation of the cellular cholesterol metabolism of various signalling molecules (Src-like kinases, H-Ras, endothelial nitric-oxide synthase, and G proteins); these molecules lead to effective communication between extracellular signals and the interior of the cell [10]. Cav-1 is predominately found in terminally differentiated cells, such as adipocytes, endothelia and smooth muscle cells, and type I pneumocytes [11]; additionally, it has been identified in skeletal muscles [12] and the liver [13, 14].

Cav-1 deficiency is related to impaired mitochondrial function, free cholesterol accumulation in mitochondrial membranes, increased membrane condensation, a reduction of the respiratory chain, and accumulation of reactive oxygen species [15].

However, to our knowledge, there are no data explaining the mechanism responsible for the cholesterol depletion in mitochondria following exercise. Therefore, the aim of this study was to investigate the effect of prolonged swimming on mitochondrial Cav-1 and cholesterol concentrations as well as the induction of changes in mitochondrial swelling in rat skeletal muscles and livers. Based on our previous data [2], we hypothesize that both exercise-induced changes in the mitochondrial swelling and mitochondrial cholesterol levels will occur with concomitant increases in Cav-1 concentrations in the skeletal muscle mitochondria but not in the liver.

## 2. Materials and Methods

### 2.1. Materials/Reagents

All chemicals were purchased from Sigma (St. Louis, MO, USA), with the exception of BSA (Merck, Darmstadt, Germany). The cholesterol assay kit was generously donated by CHEMA Diagnostica (Monsano, Italy).

### 2.2. Animals

#### 2.2.1. Animal Care

Male Wistar rats (*n* = 12) weighing 400–450 g were housed in an environmentally controlled room (23 ± 1°C with a 12 h light-dark cycle); the rats received standard rat chow and water ad libitum. Experiments were conducted in accordance with the principles of the UK legislation and were approved by the Local Ethics Committee of the Gdansk Medical University (consent no. 13/2007). The animals were anesthetized by intraperitoneal injection of ketamine and xylazine (90 and 10 mg·kg^−1^, resp.). Skeletal muscles (m. quadriceps) and livers of the anesthetized animals were rapidly removed, weighed, and immersed in an ice-cold isolation buffer (0.07 M sucrose, 0.23 M mannitol, 0.003 M HEPES, and 0.001 M EGTA; pH 7.4 for the liver and 0.17 M sucrose, 0.075 M KCl, 0.05 M Tris-base, 0.001 M KH_2_PO_4_, 0.005 M MgCl_2_, and 0.001 M EGTA; pH 7.4 for muscle) for crude mitochondria isolation.

#### 2.2.2. Exercise Protocol

The rats were prepared for the experiments and exercise tests using the previously described methods [2, 16]. The rats were randomly divided into sedentary controls (C) and long-lasting endurance exercise (E) groups. Before the experiments, the animals in the E group were trained to reduce the stress of swimming. Each day during the preparatory procedure, the rats swam for 30 min in water at 35°C. On the first day, the rats swam without any additional weight. On the second, third, and fourth days, the rats swam burdened with 1, 2, and 3% of their body weight, respectively, on their tails. On the fifth day, exercise testing was performed in the E group of rats, which consisted of 3 h of prolonged swimming in 35°C water, burdened with 3% of their body weight. The rats were euthanized (as described in Section 2.2.1) immediately after completing their protocol. As we previously demonstrated, the temperature of the water and the preparatory procedure prior to the exercise test did not influence the studied parameters [1].

### 2.3. Isolation of Rat Mitochondria 

#### 2.3.1. Rat Liver Mitochondria (RLM)

The liver mitochondria were isolated, as previously described by Broekemeier et al. [17] with slight modification. The liver was rapidly removed, weighed, and placed in an ice-cold mitochondrial buffer A (mM: 230 mannitol, 70 sucrose, 3 HEPES, and 0.1 EGTA, pH 7.4); it was then rinsed three times. The liver was then minced with scissors and homogenized using a Teflon pestle homogenizer in buffer B (buffer B = buffer A + 0.1% BSA). The homogenate was centrifuged at 700 ×g for 10 min. The supernatant was decanted and centrifuged at 7000 ×g for 10 min. The pellet was resuspended in 40 mL of suspension (buffer C = buffer A without 1 mM EGTA) and centrifuged again at 7000 ×g for 10 min. This step was repeated three times. The final mitochondrial pellet was resuspended in 0.5 mL of buffer C.

#### 2.3.2. Rat Quadriceps Mitochondria (RQM)

The skeletal muscle mitochondria were isolated, as previously described by Fontaine et al. [18] with slight modifications. The quadriceps muscle was dissected from the surrounding connective tissue, rapidly removed, trimmed clean of visible connective tissue, weighed, and placed in 10 mL of ice-cold mitochondrial isolation buffer A (mM: 150 sucrose, 75 KCl, 50 Tris base, 1 KH_2_PO_4_, 5 MgCl_2_, 1 EGTA, and 0.2% BSA, pH 7.4). Muscles were minced with scissors, incubated for 1 min with Nagarse protease (10 mL of isolation buffer per 1 g of tissue, supplemented with Nagarse (0.2 mg mL^−1^)), and homogenized using a Teflon pestle homogenizer. The homogenate volume was increased to 40 mL by adding cold isolation buffer, which was then centrifuged at 700 ×g for 10 min. The supernatant was decanted and centrifuged at 10 000 ×g for 10 min. The pellet was resuspended in 40 mL of suspension buffer B (mM: 250 sucrose, 10 Tris-base, and 0.05 EGTA, pH 7.4) and centrifuged at 10 000 ×g for 10 min. The final mitochondrial pellet was resuspended in buffer B (0.25 *μ*L of buffer B per 1 g of muscle mass).

All steps were performed at 4°C.

### 2.4. Swelling of the Mitochondria

The measurement of RLM and RQM swelling was spectrophotometrically performed, as previously described for the liver by Crouser et al. [19] and for the skeletal muscle by Csukly et al. [20]. Liver mitochondria were incubated at 25°C in a medium containing (in mM) 230 mannitol, 70 sucrose, 2.0 K_2_HPO_4_, and 3.0 HEPES, pH 7.4. RQM were incubated at the same temperature in a medium containing (in mM) 250 sucrose, 10 MOPS, 10 K_2_HPO_4_, and 10 Tris-HCl, pH 7.3.

CaCl_2_ (100 *μ*M) was used as a mitochondrial permeability transition pore (mPTP) opening inducer and cyclosporin A (CSA, 1 *μ*M) was used as an mPTP opening inhibitor. 1 mg of mitochondria was added to the appropriate buffer, which was supplemented with 5 mM succinate and 1 *μ*M rotenone.

The swelling curves were recorded at 540 nm. Cuvettes, containing the mitochondrial suspension, were kept at 25°C.

The greater decrease in absorbance is related to greater susceptibility of mitochondria to calcium chloride-induced mitochondrial swelling. Such deenergized and inactivated mitochondria are rapidly and physiologically less resistant to opening mPTP, which precedes the process of cell death.

### 2.5. Cholesterol Estimation

Total lipids were extracted from the mitochondria normalized to the mitochondrial protein concentration. The cholesterol content in extracts was measured using the CHEMA Diagnostica cholesterol assay kit with a standard cholesterol solution as a reference. Total lipids extraction and cholesterol measurement procedures were performed as previously described [21].

### 2.6. Determination of Caveolin-1 Concentration in Liver and Quadriceps Crude Mitochondria

The concentration of caveolin-1 was measured using the Cloud-Clone Corporation Caveolin-1 ELISA Kit (Category number SEA214Ra) according to the manufacturer's instructions.

### 2.7. Oxidative Stress Parameters in Rat Liver and Quadriceps Crude Mitochondria

The carbonyl groups [22] and lipid dienes [23] were measured in crude mitochondria isolated from the livers and quadriceps of the C and E rat groups. The values of the carbonyl groups are expressed as nmol·mg of protein-1, and the lipid dienes are expressed as an oxidation index 233/215.

### 2.8. Protein Measurement

The protein content was measured using the method described by Lowry et al. [24] with BSA as the standard.

### 2.9. Data Analysis

Statistical analyses were performed using a software package (Statistica v. 10.0, StatSoft Inc., Tulsa, OK, USA). The results are expressed as the mean ± standard error (SE). The differences between the means were tested using the unpaired Student's *t*-test. The results were statistically significant when *P* < 0.05.

## 3. Results

### 3.1. Mitochondrial Cholesterol Content of Quadriceps Muscles and Livers in the Control and Exercised Groups of Rats

**Figure 1:**
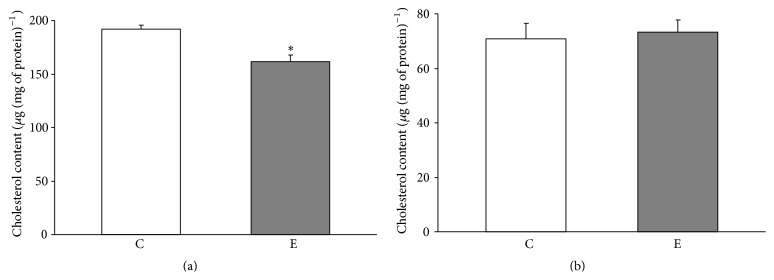
Mitochondrial cholesterol levels in controlled and exercised animals. Cholesterol was measured in crude mitochondria isolated from the quadriceps (a) and liver (b). The data are presented as the means ± SE. ^*∗*^ There was a significant difference between the E (exercise) versus C (control) in the Quadriceps mitochondria ^*∗*^ *P* = 0.001 versus the control rats (*n* = 6 in each group).

The prolonged swimming protocol caused a significant drop in the cholesterol level in crude mitochondria of the quadriceps muscle. The mitochondria isolated from the skeletal muscle of exercised rats had approximately 84% of the cholesterol compared with control rats (Figure 1(a)). No significant changes in the cholesterol levels were observed in crude mitochondria isolated from liver (C versus E group, Figure 1(b)).

### 3.2. Swelling of Mitochondria

**Figure 2:**
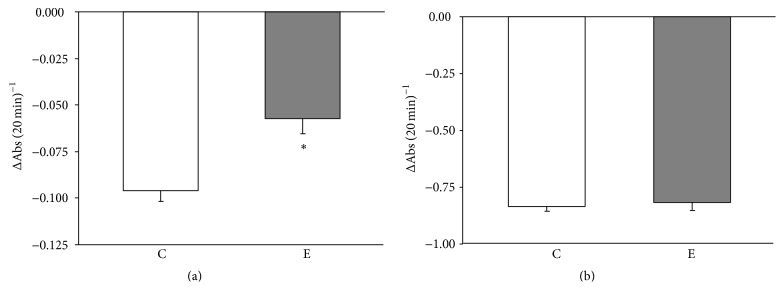
Mitochondrial swelling in controlled and exercised animals. The calcium chloride-induced mitochondrial swelling was spectrophotometrically assessed in the quadriceps (a) and liver (b) crude mitochondria. The data are presented as the means ± SE. ^*∗*^ There was a significant difference between the E (exercise) versus C (control) in the quadriceps mitochondria ^*∗*^ *P* = 0.002 versus the control rats (*n* = 6 in each group).

We determined whether mitochondrial cholesterol depletion was associated with mitochondrial swelling. The calcium chloride-induced mitochondrial swelling was significantly lower in the rat quadriceps muscle of mitochondria isolated after exercise when compared with the control (Figure 2(a)). The inhibitory effects of 1 *μ*M CSA (data not shown) were observed. No changes in the mitochondrial swelling were observed in the liver mitochondria (Figure 2(b)).

### 3.3. Content of the Caveolin-1 in Control and Exercised Quadriceps and Liver Mitochondria

To verify whether the depletion of the mitochondrial cholesterol level after exercise is related to changes in Cav, the concentration of Cav-1 was determined in crude mitochondria of the C and E groups.

**Figure 3:**
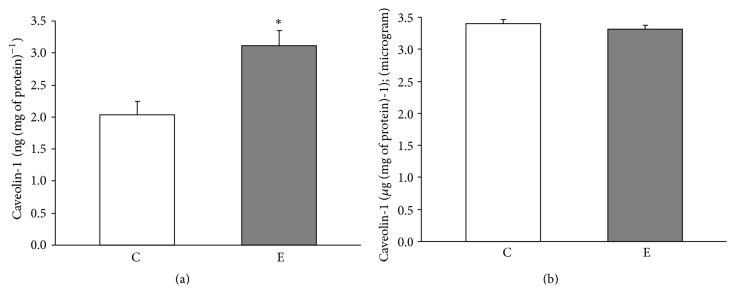
Mitochondrial caveolin-1 levels in controlled and exercised animals. Caveolin-1 level was measured in crude mitochondria isolated from the quadriceps (a) and liver (b). The data are presented as the means ± SE. ^*∗*^ There was a significant difference between the E (exercise) versus C (control) in the quadriceps mitochondria ^*∗*^ *P* = 0.006 versus the control rats (*n* = 6 in each group).

**Figure 4:**
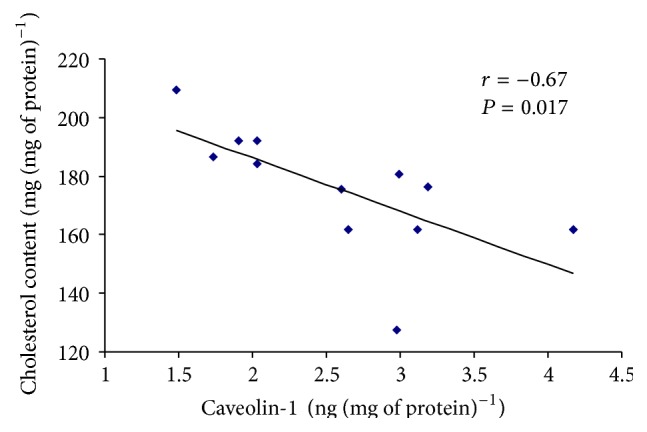
Correlation between caveolin-1 and cholesterol levels in quadriceps crude mitochondria. A Pearson product-moment correlation coefficient was computed to assess the relationship between the caveolin-1 and cholesterol level in the crude mitochondria isolated from the quadriceps. There was a large, negative correlation between the two variables, *r* = −0.67, *P* = 0.017.

Prolonged swimming caused a significant increase in the content of Cav-1 in the quadriceps of crude mitochondria (Figure 3(a)). No changes in the Cav-1 content were observed in crude mitochondria isolated from liver (Figure 3(b)). The relationship between cholesterol and Cav-1 content in the crude quadriceps muscle mitochondria is presented in Figure 4.

### 3.4. Prolonged Swimming Induces Oxidative Stress in Livers but Not in Skeletal Muscles

To verify that exercise was able to induce oxidative stress, protein carbonyl groups and lipid dienes were measured in the liver and quadriceps muscle crude mitochondria of the C and E groups of rats. Significantly higher oxidative stress parameters were only observed in the E group of crude mitochondria in the liver (Table 1).

## 4. Discussion

In the present study, we demonstrate that adaptive responses to prolonged exercise are related to increased content of Cav-1 in isolated quadriceps muscle mitochondria; moreover, there is concomitant depletion of mitochondrial cholesterol and increased resistance to Ca^2+^ induced swelling. We did not observe similar changes in rat liver mitochondria from the exercised group, but we did observe an increase in the level of protein carbonyl groups and lipid dienes in mitochondria (markers of oxidative stress). There was an inverse correlation between mitochondrial cholesterol and Cav-1 levels in rat tissues after exercise. The data confirm our initial hypothesis that both exercise-induced changes in mitochondrial swelling and the level of cholesterol are related to increases in Cav-1 concentrations in skeletal muscle mitochondria but not in the liver. We suspect that this phenomenon refers exclusively to the contractile tissues, such as heart and skeletal muscles. The finding implies that a reduction in the cholesterol levels and an increased mitochondria sensitivity to swelling are not deleterious; furthermore, these alterations may be a result of changes in Cav-1 concentrations in the mitochondria. As we have previously indicated, these modifications are related to a dynamic physiological process that may help mitochondria adapt to stress conditions [1, 2]. Changes in mitochondrial cholesterol levels in exercised rats were previously observed in heart tissues but not in skeletal muscles. Another novelty of this study is an indication of the possible role of Cav-1 in the aforementioned process.

### 4.1. The Effect of Swimming Exercise on Cav-1 and Cholesterol Levels in Liver and Skeletal Muscle Mitochondria

To our knowledge, there are no data that show changes in cholesterol and Cav-1 levels in liver and skeletal muscle mitochondria after exercise. However, Park and colleagues showed that there are changes in the Cav-1 concentrations in skeletal muscles [25] and in rat brain tissues following training [26]. The Cav-1 in the skeletal muscle is a fibre and exercise-specific [12].

Our data demonstrate that prolonged swimming reduced cholesterol levels and increased Cav-1 concentrations in rat skeletal muscle mitochondria. We did not observe such changes in the hepatic mitochondria, which leads us to suspect that this effect is specific to contractile tissues. Furthermore, there were exercise-induced changes in the mitochondrial Cav-1 and cholesterol content in skeletal muscles but not in the liver; this may have resulted from different responses in these tissues during oxidative stress.

Recently, we reported that modifications in mitochondrial function depend on the degree of depletion of cholesterol. This may be comparable to the changes observed after exercise. These changes did not impair the mitochondrial bioenergetics; furthermore, the changes positively inhibited the mitochondrial swelling, likely by remodelling the lipid microdomains [2]. However, in the* in vitro* study, adding 4% methyl-beta-cyclodextrin (a known cholesterol chelator) resulted in a lowering of the cholesterol in mitochondria below the range observed after exercise; additionally, there was a decline in mitochondrial function, which led to changes in the mitochondrial configuration state [27]. Thus, an appropriate level of cholesterol in mitochondria is necessary to maintain their function. Both too high and too low mitochondrial cholesterol levels lower the oxidative phosphorylation. This may be the main reason that mitochondrial dysfunction is related to a decrease in ATP synthesis, ATP hydrolysis (respiration in mitochondria), and impaired ADP/ATP exchange (for review, see [28]).

It has been shown that mitochondria are cholesterol-sensitive organelles, but little is known about the regulation of their cholesterol influx/efflux [29]. Cav-1, which is expressed abundantly in lipid rafts of many cells and organelles membranes [10], seems to be an excellent candidate for the cholesterol regulator in mitochondria. In fact, this protein plays an important role in the cholesterol transport inside the cell and in organelles, particularly mitochondria [15]. Bosch and coworkers demonstrated,* in vitro*, that mouse embryonic fibroblast cells from Cav-1^−/−^ mice exhibited free cholesterol accumulation in mitochondrial membranes, increased membrane condensation, reduced efficiency of the respiratory chain, and intrinsic antioxidant defences when compared with wild-type mice [15]. It was shown that changes in the level of cholesterol in the mitochondria are dependent on the presence of caveolin; this, therefore, affects the function of mitochondria and antioxidant status of the cells. These observations have been supported by Chen et al. [30], who reported that Cav-1 is a modifier of the hepatic mitochondrial respiratory chain function, antioxidant enzyme defence system, and mitochondrial biogenesis under hypercholesterolemia-induced oxidative stress.

### 4.2. The Effect of Changes in Caveolin and Cholesterol Levels on Cellular and Mitochondrial Oxidative Stress and Mitochondrial Swelling

There is an abundance of data which indicate that alternations in caveolin and cholesterol levels in the cell lead to oxidative stress. It was shown that elevated mitochondrial cholesterol levels lead to reduced 2-oxoglutarate transport and an impaired glutathione (GSH) import into mitochondria [15, 31–38]. The reduction of GSH levels is associated with increased oxidative stress [15], which is a key element that initiates mitochondrial swelling [39]. Recently, we demonstrated that the reduction in the mitochondrial cholesterol pool from exercised hearts was not associated with oxidative stress in mitochondria, despite the p66Shc phosphorylation; additionally, there was no induction of cellular apoptosis [1]. The data presented here also support this observation. Prolonged swimming was able to induce oxidative stress; additionally, there was a decrease in the ability to inhibit the mitochondrial swelling in rat livers only. On the contrary, there was no oxidative stress in skeletal muscle mitochondria, where the Cav-1 concentration was higher and the cholesterol level was decreased. Moreover, these organelles were more resistant to the calcium-induced swelling.

Studies in the changes in Cav-1 and cholesterol concentrations in mitochondria seem to be important for understanding the physiological and pathological mechanisms that occur in mitochondria in living organism.

**Figure 5:**
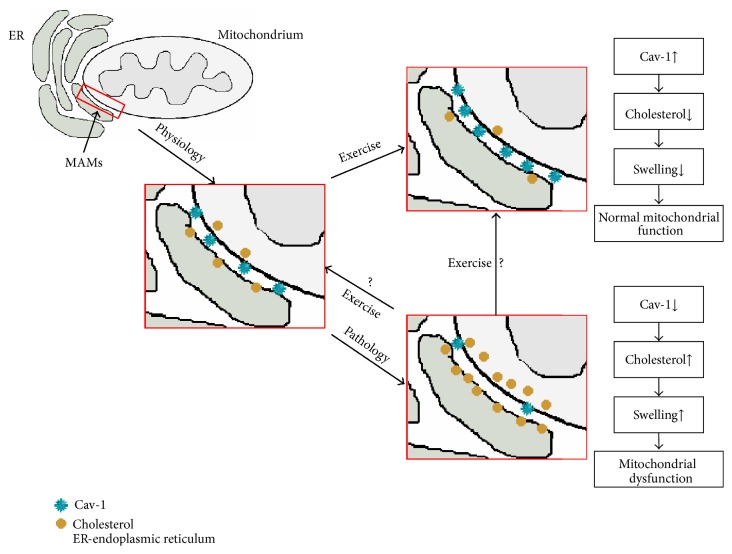
The Cav-1-dependent hypothesis of mitoprotection under exercise conditions. Under physiological conditions: appropriate levels of Cav-1 and cholesterol concentrations are presented in the mitochondria of contractile tissues. Under pathological conditions in crude mitochondria, decreased Cav-1, increased cholesterol concentrations, and mitochondrial swelling are observed. Exercise induces the opposite effects; for example, increased Cav-1 levels and decreased cholesterol levels in mitochondria lead to the inhibition of mitochondria swelling.

The mechanism behind chronic exercise on the above phenomena remains to be resolved. Based on our previously published results and the results of others [1, 2, 15, 27, 39], it can be suggested that the Cav-1-dependent hypothesis of mitoprotection is under the influence of exercising conditions (Figure 5). Under physiological conditions, appropriate levels of Cav-1 and cholesterol concentrations are present in the mitochondria of contractile tissues. However, in pathological situations, such as ischaemia/reperfusion, decreased Cav-1 and increased cholesterol concentrations in mitochondria are observed. These changes may be associated with remodelling of the mitochondrial raft-like microdomains, mitochondrial swelling, and its respective dysfunction. Exercise induces the opposite effects: for example, increased Cav-1 levels and decreased cholesterol levels lead to the inhibition of mitochondrial swelling. The mitoprotective effect of exercise is possible only when oxidative stress does not occur in the mitochondria.

The presented data indicate exercise-induced modification in the mitochondrial cholesterol content specific to contractile tissue, which may have resulted from various response to oxidative stress. Moreover, the possible role of Cav-1 on mitochondrial cholesterol depletion following exercise may be fundamental for understanding the mitoprotective mechanism during stressful conditions. Further investigations are required to clarify the role of cholesterol and Cav-1 changes in mitochondria of different tissues following exercise and to explain whether exercise might reverse the mitochondria from a pathological to physiological state via a Cav-1-dependent mechanism.

## Figures and Tables

**Table 1:** Oxidative stress parameters (carbonyl groups and lipid dienes) in Quadriceps and Liver mitochondria of the C and E groups of rats.

	Quadriceps mitochondria		Liver mitochondria	
	C	E	C	E
Carbonyl groups (nmol (mg of protein)^−1^)	1.460 ± 0.08	1.364 ± 0.12	1.125 ± 0.07	1.732 ± 0.12^*∗*^
Lipid dienes (oxidation index (233/215))	0.172 ± 0.003	0.169 ± 0.004	0.230 ± 0.005	0.260 ± 0.005^#^

The mean values ± SE are expressed as nmol (mg of protein)^−1^ (for carbonyl groups) and oxidation index 233/215 (lipid dienes). ^*∗*^ *P* = 0.0008, ^#^ *P* = 0.0007. The E (exercise) group is significantly different from the C (control) group (*n* = 6 in each group).
